# Blood kynurenine pathway metabolites show limited diagnostic utility for tuberculosis among adults living with human immunodeficiency virus and children in Uganda

**DOI:** 10.1016/j.ijregi.2026.100994

**Published:** 2026-09-07

**Authors:** George W. Kasule, Joachim Nsubuga, René Lutter, Derrick Semugenze, Frederic Vaz, Marion Kolader, Frank Cobelens, Patricia Mwachan, James Sserubiri, Youssra Jamal, Moses Joloba, Alberto L. García-Basteiro, Willy Ssengooba, Sabine Hermans, Stool4TB Global Partnership

**Affiliations:** 1 Department of Medical Microbiology, College of Health Sciences, Makerere University, Kampala, Uganda; 2 Barcelona Institute for Global Health (ISGlobal), Hospital Clínic, Universitat de Barcelona, Barcelona, Spain; 3 National Tuberculosis Reference Laboratory, National Tuberculosis and Leprosy Programme, Ministry of Health, Kampala, Uganda; 4 Department of Respiratory Diseases, Amsterdam UMC, Location University of Amsterdam, Amsterdam, The Netherlands; 5 Department of Laboratory Medicine, Laboratory Genetic Metabolic Diseases, Emma Children's Hospital, Amsterdam UMC, Location University of Amsterdam, Amsterdam, The Netherlands; 6 Amsterdam Gastroenterology Endocrinology Metabolism, Inborn Errors of Metabolism, Amsterdam UMC location University of Amsterdam, Amsterdam, The Netherlands; 7 Core Facility Metabolomics, Amsterdam UMC, Location University of Amsterdam, Amsterdam, The Netherlands; 8 Department of Medical Microbiology and Infection Prevention, Amsterdam UMC, Location University of Amsterdam, Amsterdam, The Netherlands; 9 Department of Global Health, Amsterdam Institute for Global Health and Development, Amsterdam UMC, Location University of Amsterdam, Amsterdam, The Netherlands; 10 Tuberculosis Research Group, Centro de Investigação em Saúde de Manhiça (CISM), Maputo, Mozambique; 11 Makerere University Lung Institute (MLI), Makerere University, Kampala, Uganda; 12 Centre for Tropical Medicine and Travel Medicine, Department of Infectious Diseases, Amsterdam Public Health – Global Health, Amsterdam Institute for Immunity and Infectious Diseases, Amsterdam UMC, Location University of Amsterdam, Amsterdam, The Netherlands

**Keywords:** Kynurenine pathway, Indoleamine 2,3-dioxygenase, Tuberculosis, PWH, Children, Blood

## Abstract

**Objectives:**

Traditional sputum-based diagnostics often demonstrate suboptimal diagnostic performance in people living with human immunodeficiency virus and children presenting with paucibacillary tuberculosis (TB). This study evaluated the diagnostic accuracy of circulating kynurenine-pathway metabolites against an authentic symptomatic control cohort in Uganda.

**Methods:**

Tryptophan, kynurenine, and the kynurenine-to-tryptophan ratio were quantified via ultra-performance liquid chromatography-tandem mass spectrometry in 70 adult people living with human immunodeficiency virus and 23 children presenting with presumptive TB.

**Results:**

Median biomarker concentrations demonstrated no statistically significant differences between participants with laboratory-confirmed TB and symptomatic controls. The kynurenine-to-tryptophan ratio yielded an area under the curve of 0.51 (95% confidence interval: 0.34–0.69) in adults and 0.62 (95% confidence interval: 0.36–0.87) in children, indicating limited diagnostic discrimination with substantial uncertainty, particularly in the small pediatric cohort. At the evaluated cutoff points, none of the biomarkers met the minimum sensitivity and specificity requirements defined by the World Health Organization target product profiles for diagnostic and screening tests.

**Conclusion:**

Blood kynurenine pathway metabolites demonstrated limited clinical utility as standalone diagnostic biomarkers for active TB in these vulnerable populations. In addition, larger diagnostic performance studies are needed to address the uncertainty associated with the small sample size and to further validate our findings.

## Highlights

• The study evaluated blood biomarkers in adults with human immunodeficiency virus and children with presumptive tuberculosis.
• Blood biomarkers did not differentiate tuberculosis from symptomatic controls.
• Symptomatic controls provided real-world diagnostic assessment.
• Host-response biomarkers showed limited standalone tuberculosis diagnostic utility.
• Future validation studies should prioritize clinically representative controls.

## Summary

In this tuberculosis laboratory diagnostic evaluation study, blood tryptophan, kynurenine, and kynurenine-to-tryptophan ratios demonstrate poor diagnostic accuracy and have limited utility as standalone biomarkers to distinguish laboratory-confirmed tuberculosis from symptomatic respiratory controls among Ugandan children and adults living with human immunodeficiency virus.

## Background

In 2024, tuberculosis (TB) reclaimed its status as the leading cause of death from a single infectious pathogen [1]. Accurate diagnosis in people living with human immunodeficiency virus (PWH) and younger children remains a profound public health challenge because of the predominantly paucibacillary nature of disease, which renders conventional sputum-based molecular and microbiological diagnostics significantly less sensitive [1]. Consequently, a critical need persists for non-sputum-based diagnostic approaches.

Circulating blood biomarkers related to the host kynurenine pathway, regulated by the interferon-gamma-inducible enzyme indoleamine 2,3-dioxygenase (IDO), have emerged as a promising target [2]. Elevated host IDO activity, measured via the kynurenine-to-tryptophan (Kyn/Trp) ratio, is a well-documented feature of active TB and other infections [[3], [4], [5], [6], [7], [8]]. However, the majority of prior TB biomarker evaluation studies have been conducted within highly idealized populations, frequently utilizing completely healthy, asymptomatic volunteers as controls.

This diagnostic performance evaluation measured absolute blood tryptophan, kynurenine, and the resulting Kyn/Trp ratio within adult PWH and pediatric cohorts presenting with presumptive TB in Uganda to determine whether these host-response immunometabolic markers can reliably differentiate participants with laboratory-confirmed TB from symptomatic controls.

## Methods

### Study design and participants

This diagnostic accuracy study was nested within the multicenter Stool4TB parent study and implemented at the Ugandan site between December 2021 and August 2024 among participants with presumptive TB seeking care at primary healthcare facilities. Written informed consent or assent was systematically obtained [9]. We did not perform an *a priori* statistically powered sample size calculation; instead, the substudy included participants from the parent study [9] who consented to participate and had available plasma or serum samples.

Eligibility criteria were based on those of the parent study for pediatric participants (aged <8 years) and adult PWH (aged ≥15 years), with a few modifications restricted to include children aged 3 to 8 years.

Pediatric participants were categorized into two analytical groups following National Institutes of Health guidelines [10]: microbiologically confirmed TB (confirmed TB), defined as a positive result on at least one sputum culture, sputum Xpert Ultra, or stool Xpert Ultra assay; and unlikely TB (No-TB) controls, defined as individuals without bacteriological confirmation and in whom unconfirmed TB criteria were not met [10].

The adult PWH cohort was categorized into confirmed TB, defined as a positive result on at least one baseline sputum culture, sputum Xpert Ultra, or urine lateral flow lipoarabinomannan (LAM) assay; and No-TB, defined as negative results across all baseline diagnostic tests above [9]. We included all adults with confirmed TB and controls with available plasma collected by the parent study, whereas children with confirmed TB and controls with serum samples were individually matched by age and sex at a 1:2 ratio. One control serum sample was not analysed in the laboratory and hence excluded from data analysis.

### Laboratory and statistical analysis

Biological specimens collected in the parent study included expectorated or induced sputum for Xpert® MTB/RIF Ultra and culture (hereafter, Ultra; Cepheid, Sunnyvale, CA, USA); stool for Xpert Ultra; urine for Determine^TM^ TB-LAM Antigen testing (Abbott Laboratories, Waltham, MA, USA); and blood for plasma and/or serum separation and testing for human immunodeficiency virus (HIV) and CD4 cell count [9] and analysis previously described [11] using Pima™ CD4 Analyzer (Abbott Laboratories, Waltham, MA, USA) in the respective enrolment sites. Separated plasma and serum aliquots were stored at −80°C and shipped frozen to the Amsterdam University Medical Centre (Amsterdam UMC) for target analyte quantification via ultra-performance liquid chromatography-tandem mass spectrometry using previously validated protocols [6]. Laboratory personnel performing tests were blinded to the participants’ clinical information and reference standard results.

Absolute metabolite concentrations were compared using the nonparametric Mann–Whitney *U* test. Overall diagnostic performance was assessed via receiver operating characteristic curve analysis to derive the area under the curve (AUC) and optimal operational cutoff values showing sensitivity and specificity, which were compared against the minimum criteria specified within the official World Health Organization target product profiles [12,13]. An exploratory *post hoc* analysis included the AUC of the inverted tryptophan and was not part of the prespecified primary analysis. This was performed to reflect the depletion of tryptophan associated with its conversion to kynurenine; the inverse of the measured tryptophan (1/tryptophan) was also evaluated as an exploratory marker. In addition, a *post hoc* analysis for multiple testing was conducted. Statistical significance was defined as a two-tailed *P*-value ≤0.05, and computational analyses were executed using Stata version 17 (College Station, TX: StataCorp LLC).

## Results

The final analytical datasets included plasma from 70 adult PWH (19 with confirmed TB, 51 No-TB controls) and serum from 23 children (eight with confirmed TB, 15 No-TB controls). Within the adult PWH cohort, the median age was 37.5 years, 62.9% were female, 100.0% were receiving antiretroviral therapy at enrollment, and 82.9% presented with preserved CD4 cell counts >200 cells/µL. The pediatric cohort exhibited a median age of 5.4 years, and 91.3% were HIV-negative (Table 1).

**Table 1:** Participants’ demographic data categorized by children and adults.

	Children		Adult PWH	
	Microbiologically confirmed TB, n = 8 (34.8%)	No-TB, n = 15 (65.2%)	Microbiologically confirmed, TB n = 19 (27.1%)	No-TB, n = 51 (72.9%)
**Demographic and epidemiological data**				
**Gender**	n (%)		n (%)	
Female	3 (37.5)	5 (33.3)	15 (78.9)	29 (56.9)
Male	5 (62.5)	10 (66.7)	4 (21.1)	22 (43.1)
**Age (years)**				
Median (IQR)	5.3 (4.7-6.8)	5.9 (4.5-6.5)	27.0 (24.0-35.5)	41.9 (33.0-53.9)
**Clinical and radiological data**				
**Cough**				
Yes	8 (100.0)	15 (100.0)	19 (100.0)	51 (100.0)
**Fever**				
Yes	6 (75.0)	9 (60.0)	11 (57.9)	28 (54.9)
No	2 (25.0)	6 (40.0)	8 (42.1)	23 (45.1)
**CD4 count**^a^				
≤200 cells/µL			2 (10.5)	6 (11.8)
>200 cells/µL			16 (84.2)	42 (82.4)
Not done			1 (5.3)	3 (5.9)
**Nutritional status**				

	Children		Adult PWH	
Category	Microbiologically confirmed TB, n = 8 (34.8%)	No-TB, n = 15 (65.2%)	Microbiologically confirmed, TB n = 19 (27.1%)	No-TB, n = 51 (72.9%)

Underweight	3 (37.5)	4 (26.7)	3 (15.8)	6 (11.8)
Healthy weight	5 (62.5)	9 (60.0)	11 (57.9)	32 (62.7)
Overweight )	0 (0.0)	2 (13.3)	4 (21.1)	9 (17.6)
Obesity	0 (0.0)	0 (0.0)	1 (5.3)	4 (7.8)

Data are n (%) unless otherwise indicated. Children with microbiologically confirmed TB are positive on at least one sputum culture, Ultra or stool Ultra test, and adults are positive on at least one sputum culture, Ultra or urine LAM test. Children in the No-TB group (non-microbiologically confirmed) are without bacteriological confirmation and do not meet the unconfirmed tuberculosis criteria according to the NIH classification; adults are negative on all laboratory confirmatory tests. Nutritional status in children was determined by using the WHO BMI-for-age classification in children, with BMI [weight (kg)/height (m)²] assessed against the WHO BMI-for-age chart based on the gender of the child. BMI [weight (kg)/height (m)²] in adults was categorised as <18.5: underweight; 18.6—25: healthy weight; 25.1—30: overweight; >30: Obese.

BMI, body mass index; CD4, cluster of differentiation 4; HIV, human immunodeficiency virus; IQR, interquartile range; LAM, lipoarabinomannan; NIH, National Institutes of Health; PWH, people living with HIV; TB, tuberculosis; WHO, World Health Organization.

a No HIV positive children (2/23) had CD4 count results. Percentages may not total 100% due to rounding.

Absolute median concentrations of tryptophan, kynurenine (expressed in µM), and the resulting Kyn/Trp ratio demonstrated no statistically significant differences between participants with confirmed TB and No-TB controls in either cohort (Table 2)*.* Additionally, we conducted sensitivity analyses using Bonferroni and Holm-Bonferroni adjustments for multiple testing to assess the potential risk of type I error. All adjusted *P*-values remained nonsignificant, ranging from 0.54 to 1.00. As none of the raw observed comparisons reached the conventional threshold for statistical significance (all reported raw *P*-values >0.05; Table 2), adjustment for multiple testing did not change the interpretation of the findings or the conclusions.

**Table 2:** Comparison of blood tryptophan and kynurenine concentrations and kynurenine-to-tryptophan ratio biomarkers between microbiologically confirmed and non-microbiologically confirmed TB groups (No-TB) in adult PWH and children.

A. Metabolite concentrations (µM) and Kyn/Trp ratio							
Adults	Microbiologically confirmed TB n = 19		No-TB n = 51			Overall n = 70	
	Median (IQR)	Mean (SD)	Median (IQR)	Mean (SD)	*P*-value	Median (IQR)	Mean (SD)
Tryptophan	40.4 (28.1-47.9)	39.2 (13.9)	41.3 (33.4-50.2)	40.9 (10.2)	0.87	41.1 (32.8-48.9)	40.4 (11.3)
Kynurenine	2.0 (1.7-2.5)	2.4 (1.3)	2.0 (1.7-2.4)	2.2 (0.7)	0.95	2.0 (1.7-2.4)	2.2 (0.9)
Kyn/Trp ratio	0.05 (0.04-0.10)	0.07 (0.06)	0.05 (0.04-0.07)	0.06 (0.03)	0.86	0.05 (0.04-0.07)	0.06 (0.04)

**Children**	**Microbiologically confirmed TB n = 8**		**No-TB n = 15**			**Overall n = 23**	
	Median (IQR)	Mean (SD)	Median (IQR)	Mean (SD)	*P*-value	Median (IQR)	Mean (SD)

Tryptophan	43.3 (35.5-50.2)	42.4 (10.5)	48.1 (42.1-55.2)	49.3 (9.9)	0.18	47.3 (40.9-53.0)	46.9 (10.4)
Kynurenine	2.0 (1.8-2.6)	2.3 (0.8)	2.1 (1.9-2.6)	2.3 (0.5)	0.54	2.1 (1.9-2.6)	2.3 (0.63)
Kyn/Trp ratio	0.05 (0.04-0.08)	0.06 (0.02)	0.04 (0.03-0.06)	0.05 (0.01)	0.36	0.04 (0.04-0.07)	0.05 (0.02)
**B. Area under the curve (95% CI)**							

**Adults n = 70**				**Children n = 23**			

Tryptophan	0.49 (0.32-0.65)			0.33 (0.08-0.57)			
Inverted tryptophan	0.51 (0.35-0.68)			0.67 (0.42-0.92)			
Kynurenine	0.50 (0.35-0.65)			0.42 (0.14-0.70)			
Kyn/Trp ratio	0.51 (0.34-0.69)			0.62 (0.36-0.87)			
**C. Sensitivity and specificity at selected cutoff points**							

	**Adults (Sensitivity, Specificity)**			**Children (Sensitivity, Specificity)**			

Tryptophan	≥28.11 (78.9%-11.8%)			≥43.7 (50.0%-40.0%)			
	≥44.71 (42.1%-64.7%)			≥52.2 (25.0%-60.0%)			
Kynurenine	≥1.55 (89.5%-13.7%)			≥1.7 (87.5%-6.7%)			
	≥2.36 (31.6%-68.6%)			≥2.6 (25.0%-60.0%)			
Kyn/Trp ratio	≥0.05 (47.4%-51.0%)			≥0.05 (50.0%-66.7%)			
	≥0.07 (31.6%-80.4%)			≥0.08 (25.0%-86.7%)			

Children with microbiologically confirmed TB are positive on at least one sputum culture, Ultra or stool Ultra test, and adults are positive on at least one sputum culture, Ultra or urine LAM test. Children in the No-TB group (non-microbiologically confirmed) are without bacteriological confirmation and do not meet the unconfirmed tuberculosis criteria according to the NIH classification; adults are negative on all laboratory confirmatory tests.

A. Concentrations measured in µM (Kyn/Trp ratios are unitless); B. Area under the receiver operating characteristic curve; and C. Selected cutoffs around the WHO TPPs for TB detection by a non-sputum, low complexity assay of test of minimum sensitivity, 80.0% and specificity, 98% (Kohli et al., 2025).

CI, confidence interval; IQR, interquartile range; Kyn/Trp, kynurenine-to-tryptophan; LAM, lipoarabinomannan; NIH, National Institutes of Health; PWH, people living with human immunodeficiency virus; TB, tuberculosis; TPP, target product profile; WHO, World Health Organization.

Among adult PWH, the Kyn/Trp ratio achieved an AUC of 0.51 (95% confidence interval [CI]: 0.34-0.69). Within the pediatric cohort, the Kyn/Trp ratio yielded a moderate AUC of 0.62 (95% CI: 0.36-0.87) (Table 2). Standalone raw tryptophan and kynurenine concentrations performed poorly across both populations (individual AUC values ranging from 0.39 to 0.50).

Evaluating the mathematically inverted tryptophan concentration (1/tryptophan) to reflect IDO-mediated depletion slightly improved the performance, yielding an AUC of 0.67 (95% CI: 0.42-0.92) in children and 0.51 (95% CI: 0.35-0.68) in adults. None of the biomarkers met the minimum thresholds defined in the World Health Organization target product profiles for decentralized screening or triage diagnostics (Table 2).

## Discussion

Our findings demonstrate that blood kynurenine metabolites carry limited clinical utility when deployed as standalone biomarkers in these populations.

Unlike previous studies that compared participants with active TB against healthy asymptomatic controls, our study evaluated these candidate signatures within clinically representative, symptomatic individuals undergoing routine programmatic clinical investigations for presumptive TB. This pragmatic design provides a realistic assessment of true diagnostic performance and indicates that previous studies likely overestimated the performance because they compared patients with active TB with healthy asymptomatic controls rather than symptomatic individuals undergoing routine TB investigation [5,6,8].

The lack of diagnostic significance observed in our adult cohort, in contrast to some previous studies [6,8] may be attributed to high antiretroviral therapy uptake (>90.0%) and preserved immune function (CD4 counts >200 cells/µL in >75.0% of participants), both of which are strongly associated with decreased baseline host IDO enzymatic activity [6,8].

Most importantly, our control group consisted exclusively of symptomatic participants whose alternative respiratory pathologies likely triggered robust host interferon-gamma-mediated IDO expression, effectively narrowing the metabolic gap between participants with active TB and clinical controls [6,8]. Another structural strength of this study was the inclusion of urine lateral flow LAM within the adult reference standard, thereby providing a more comprehensive microbiological case definition for PWH.

Within the pediatric cohort, the lower diagnostic performance observed in our study than in that of Tornheim et al. [5] likely stems from key differences in cohort selection. Our study compared participants with active TB against symptomatic controls instead of healthy controls and uninfected household contacts. Symptomatic controls are likely to harbor alternative pathologies that may trigger robust IDO activation and tryptophan degradation. This narrows the metabolic contrast between the groups and underscores the challenges of utilizing the Kyn/Trp ratio for differential diagnosis in routine clinical settings where presenting symptoms overlap. Furthermore, neither study directly analyzed population differences in factors such as nutritional status, bacillus Calmette-Guérin vaccination status, or disease severity. Because these background factors impact on baseline biomarker concentrations and host systemic responses, they may explain the contrast in our results and ought to be considered in future validation studies.

Compared to the findings in the adult cohort, our pediatric findings reflect age-related differences and population-specific factors, including immune maturation and disease pathogenesis, as primary childhood TB is characteristically noncavitary and lacks the intense localized tissue inflammation and systemic IDO activation seen in adult reactivation disease.

Furthermore, *Mycobacterium tuberculosis* can synthesize tryptophan *de novo*, allowing partial evasion of host tryptophan depletion. Consequently, circulating Kyn/Trp ratios are more likely to reflect generalized immune activation than TB-specific disease.

Key limitations included the small sample sizes and few participants with positive results across our nested cohorts, requiring our results to be interpreted with substantial uncertainty in our estimates, as indicated by the wide confidence intervals. These limitations also restricted our statistical power to evaluate the effects of advanced HIV, CD4 cell count, comorbidities, variable TB disease severity, and population differences on these biomarkers and our ability to conduct in-depth analysis of our findings. Including the urine TB-LAM assay in the adult composite reference standard may have introduced misclassification because TB-LAM-based rapid lateral flow tests have variable sensitivity and specificity. Additionally, we were unable to compare our data against the formal National Institutes of Health-defined unconfirmed TB pediatric cohort classification group, and the exclusion of children below 3 years of age reduces the direct generalizability of these findings to infant cohorts. Furthermore, evaluating multiple biomarker profiles or transformations increases the potential risk of type I error. Although none of our observed comparisons met the conventional threshold for statistical significance, and the multiple-testing sensitivity analyses did not alter the findings, this potential limitation should be considered when interpreting the exploratory results.

## Conclusion

This study demonstrates that blood tryptophan, kynurenine, and the resulting Kyn/Trp ratio exhibit limited clinical utility when deployed as standalone biomarkers for TB screening or confirmation among Ugandan adult PWH and pediatric cohorts presenting with presumptive TB. In addition, larger diagnostic performance studies are needed to address the uncertainty associated with the small sample size and to further validate our findings.

Future translational research should investigate their diagnostic performance when multiplexed into multi-marker blood-based host-response molecular signature panels rather than used as standalone assays.

## Funding

The indoleamine 2,3-dioxygenase (IDO) substudy was made possible by funding from the Mr. Willem Bakhuys Roozeboomstichting (grant number 2021-1). The parent study is part of the European and Developing Countries Clinical Trials Partnership (EDCTP) 2 and EDCTP 3 programs, supported by the European Union (grant number RIA2018D-2511-Stool4TB). This publication reflects only the authors’ views. The views and opinions of authors expressed herein do not necessarily state or reflect those of EDCTP. Part of the bench fees and reagents for mass spectrometry were procured through a travel grant awarded by HIV Research Trust, UK, to GWK.

## Role of funding source

The study funders did not have a role in the design, data collection, data analysis, data interpretation, or writing of the report.

## Ethical approval

The parent study protocol was approved by Makerere University School of Biomedical Sciences Research and Ethics Committee (SBS-2021-25) and Uganda National Council for Science and Technology (HS1615ES). The substudy was also approved by the same institutions as SBS-2023-449 and HS4344ES, respectively.

**Ethics declaration**

**Informed consent and patient details**

Written informed consent to take part in the study and to publish the article has been obtained from all participants or their legal representatives. The privacy rights of participants have been observed.

**Human and biological material**

This study did not involve organ or tissue donors. The study was strictly limited to the collection and analysis of non-tissue clinical specimens (blood, sputum, stool, and urine), no human organs or solid tissues were sourced or utilized.

**Studies in Human**

This study was performed in compliance with relevant laws, regulatory frameworks and guidelines where the research took place. This study was approved by the Makerere University School of Biomedical Sciences Research and Ethics Committee (approval no. SBS-2023-449).

## Availability of data and materials

All data generated or analyzed during this study are included in this published article.

## Consent for publication

The funders had no role in the decision to publish this manuscript. All authors consented to publish the manuscript as submitted to the journal.

## CRediT authorship contribution statement

**George W. Kasule:** Writing – review & editing, Writing – original draft, Validation, Resources, Project administration, Methodology, Investigation, Funding acquisition, Formal analysis, Data curation, Conceptualization. **Joachim Nsubuga:** Writing – review & editing, Validation, Project administration, Formal analysis, Data curation. **René Lutter:** Writing – review & editing, Validation, Supervision, Formal analysis. **Derrick Semugenze:** Writing – review & editing, Project administration, Methodology, Investigation, Formal analysis. **Frederic Vaz:** Writing – review & editing, Methodology, Formal analysis, Data curation. **Marion Kolader:** Writing – review & editing, Investigation, Formal analysis, Data curation. **Frank Cobelens:** Writing – original draft, Validation, Methodology, Funding acquisition. **Patricia Mwachan:** Writing – review & editing, Project administration, Methodology, Investigation, Formal analysis. **James Sserubiri:** Writing – review & editing, Project administration, Methodology, Investigation, Formal analysis. **Youssra Jamal:** Writing – review & editing, Methodology, Formal analysis, Data curation. **Moses Joloba:** Writing – review & editing, Supervision, Funding acquisition. **Alberto L. García-Basteiro:** Writing – review & editing, Validation, Supervision, Project administration, Methodology, Funding acquisition, Conceptualization. **Willy Ssengooba:** Writing – review & editing, Writing – original draft, Validation, Project administration, Methodology, Funding acquisition, Formal analysis, Conceptualization. **Sabine Hermans:** Writing – review & editing, Supervision, Resources, Project administration, Methodology, Funding acquisition, Conceptualization.

## Declaration of competing interest

The authors GWK, JN, RL, DS, FV, MK, FC, PM, JS, YJ, MJ, and WS declare no competing interests.

ALG-B has received grants from European and Developing Countries Clinical Trials Partnership (EDCTP), National Institutes of Health (NIH), European Research Council (ERC), and Bill & Melinda Gates Medical Research Institute (Gates MRI) in the last 36 months; received remuneration for time invested in BioNTech advisory board meetings (fair market value); is supported by Bill & Melinda Gates Foundation (BMGF) for meetings related to TB vaccines (Collaboration for TB Vaccine Discovery (CTVD) collaboration); is part of malaria vaccine trials sponsored by Sanaria, the BioNTech advisory board for TB vaccine development and the European Regimen Accelerator for Tuberculosis (ERA4TB)- Innovative Medicines Initiative, European Union advisory board; is an associate editor of the BMJ Global Health journal and an associate editor of the *The International Journal of Tuberculosis and Lung Disease* (IJTLD journal); and chaired the TB Group at the European Respiratory Society (2000-2023). SH received funding from European and Developing Countries Clinical Trials Partnership (EDCTP) 2 and3 programs, and Mr. Willem Bakhuys Roozeboomstichting.
